# Regulatory T Cell as Predictor of Intramyocardial Hemorrhage in STEMI Patients after Primary PCI

**DOI:** 10.31083/j.rcm2407205

**Published:** 2023-07-14

**Authors:** Yue Zhang, Hui Gao, Lei Liu, Shengyu Li, Bing Hua, Dihui Lan, Yi He, Jinshui Li, Hui Chen, Weiping Li, Hongwei Li

**Affiliations:** ^1^Department of Cardiology, Cardiovascular Center, Beijing Friendship Hospital, Capital Medical University, 100050 Beijing, China; ^2^Department of Radiology, Beijing Friendship Hospital, Capital Medical University, 100050 Beijing, China; ^3^Beijing Key Laboratory of Metabolic Disorder Related Cardiovascular Disease, 100191 Beijing, China; ^4^Department of Geriatrics, Beijing Friendship Hospital, Capital Medical University, 100050 Beijing, China

**Keywords:** ST-segment elevation myocardial infarction (STEMI), primary percutaneous coronary intervention (PPCI), intramyocardial hemorrhage (IMH), regulatory T cell (Treg), prediction

## Abstract

**Background:**

Intramyocardial hemorrhage (IMH) is a result of ischemia-reperfusion injury in ST-segment elevation myocardial infarction (STEMI) after primary percutaneous coronary intervention (PPCI). Despite patients with IMH show poorer prognoses, studies investigating predictors of IMH occurrence are scarce. This study firstly investigated the effectiveness of regulatory T cell (Treg), peak value of Creatine Kinase MB (pCKMB), high-sensitivity C-reactive protein (hsCRP), and left ventricular end-systolic diameter (LVESD) as predictors for IMH.

**Methods:**

In 182 STEMI patients received PPCI, predictors of IMH were analyzed by logistic regression analysis. The predictive ability of risk factors for IMH were determined by receiver operating characteristic curves, net reclassification improvement (NRI), integrated discrimination improvement (IDI) and C-index.

**Results:**

Overall, 80 patients (44.0%) developed IMH. All 4 biomarkers were independent predictors of IMH [odds ratio [OR] (95% confidence interval [CI]): 0.350 (0.202–0.606) for Treg, 1.004 (1.001–1.006) for pCKMB, 1.060 (1.022–1.100) for hsCRP, and 3.329 (1.346–8.236) for LVESD]. After propensity score matching (PSM), the biomarkers significantly predicted IMH with areas under the curve of 0.750 for Treg, 0.721 for pCKMB, 0.656 for hsCRP, 0.633 for LVESD, and 0.821 for the integrated 4-marker panel. The addition of integrated 4-marker panel to a baseline risk model had an incremental effect on the predictive value for IMH [NRI: 0.197 (0.039 to 0.356); IDI: 0.200 (0.142 to 0.259); C-index: 0.806 (0.744 to 0.869), all *p* < 0.05].

**Conclusions:**

Treg individually or in combination with pCKMB, hsCRP, and LVESD can effectively predict the existence of IMH in STEMI patients received PPCI.

**Clinical Trial Registration:**

NCT03939338.

## 1. Introduction 

Current global guidelines for ST-segment elevation myocardial infarction (STEMI) recommend primary percutaneous coronary intervention (PPCI) as the gold standard of treatment [1, 2], and PPCI restores thrombolysis in myocardial infarction flow 3 (TIMI 3) in over 90% of patients. Despite the recovery of the epicardial coronary circulation, however, additional injury caused by PPCI such as microvascular injury, also known as the no-reflow phenomenon, cannot be ignored. Studies have confirmed that up to 40% to 50% of STEMI patients underwent PPCI may experience no-reflow phenomenon, including microvascular obstruction (MVO) and Intramyocardial hemorrhage (IMH) [3, 4]. Among them, ischemic injury of capillaries leads to the occurrence of endothelial gaps and loss of integrity of capillary wall, and extensive erythrocyte extravasation leads to IMH [5, 6]. Studies demonstrated that IMH was closely related to infarct size, MVO and impaired left ventricular (LV) function, and major adverse cardiac events (MACEs) [7, 8, 9, 10, 11, 12]. Therefore, accurate diagnosis of IMH is of great importance in the clinical. According to the recommendations of the guidelines, cardiac magnetic resonance (CMR) imaging is considered the reference diagnostic method for the evaluation of IMH [1, 13]. However, we found some disadvantages of CMR that should not be ignored in the process of practice. First of all, CMR is time-consuming, and it is dangerous for STEMI patients to go for such time-consuming test without electrocardiogram monitoring in acute phase. Second, for those patients with claustrophobia or other contraindications to CMR, it is not feasible to assess IMH by CMR. Finally, CMR is expensive, which will undoubtedly increase the cost of hospitalization for patients. Therefore, considering the above reasons, we tried to find a safe, simple and effective method to predict the presence of IMH. It is well known that myocardial ischaemia/reperfusion injury (MIRI) is a common cause of no-reflow [14], in which inflammatory response plays an important role [15, 16]. Bochaton* et al*. [17] analyzed 20 consecutive patients with STEMI-PPCI and found that high-sensitivity C-reactive protein (hsCRP) and neutrophils levels were higher in patients with IMH. In addition, although the innate immune response plays an I mportant role in ischaemia-reperfusion injury (IRI), T lymphocytes including T-helper 1 (Th1), Th2, Th17 and regulatory T (Treg) cells, are also involved in the pathogenesis of IRI.

In this study, we focused on the role of Treg cells in MIRI. As far as I know, Treg can be capable of suppressing the innate immune response by inhibiting the macrophage inflammatory phenotype and neutrophil function [18], thereby playing an anti-inflammatory effect in MIRI. Previous studies have reported that Treg can ameliorate IRI in kidney and brain [19, 20]. Recent studies have also reported the protective effect of Treg in mouse MIRI [21, 22]. In this study, we analyzed the circulating Treg level and other common laboratory indicators, and hypothesized that Treg individually or in combination with other indicators, such as left ventricular end-systolic diameter (LVESD), hsCRP and the peak value of Creatine Kinase MB (pCKMB), can be used to predict the presence of IMH in STEMI patients received PPCI.

## 2. Materials and Methods

### 2.1 Study Population 

The study population for this study was identified from the Cardiovascular Center of Beijing Friendship Hospital. The data collection process was approved by the Institutional Review Board of Beijing Friendship Hospital affiliated to Capital Medical University and was in accordance with the Declaration of Helsinki. Written informed consent was obtained from all patients. The inclusion criteria for this study were STEMI patients within 12 h of symptom onset who underwent PPCI at our hospital. The exclusion criteria included (1) previous myocardial infarction or revascularization [PCI or coronary artery bypass graft (CABG)]; (2) atrial fibrillation; (3) left ventricular ejection fraction (LVEF) <40%; (4) estimated glomerular filtration rate (eGFR) <30 mL/min/1.73 $m^{2}$; (5) rheumatic immune system disease or malignant tumor; (6) acute infectious disease within nearly 3 months; (7) claustrophobia or contraindications to CMR; and (8) disagree to be included in the study. 182 patients were enrolled from October 30, 2019 to September 20, 2021.

### 2.2 Blood Samples and Data Collections

Blood samples were obtained on the following morning of the admission day, from all patients in a fasting state. A part of venous blood was prepared into peripheral blood mononuclear cells (PBMCs) by Ficoll density gradient method, and stored at –80 °C for the following flow cytometric analysis. The remaining blood sample was sent to the Central Laboratory of Beijing Friendship Hospital to be tested by professional laboratory physicians for other indicators, including hsCRP. In order to obtain the peak value of myocardial enzymes such as pCKMB, fasting venous blood was taken every morning within 5 days after PPCI to detect the levels of myocardial injury markers.

An ultrasound cardiogram was performed within 24 h after PPCI to obtain indicators of cardiac structure and function, such as LVESD and LVEF. Five to seven days after reperfusion, IMH was assessed by CMR using T2-weighted imaging.

### 2.3 Flow Cytometric Analysis of Treg

#### 2.3.1 Cell Preparation

For analysis of Treg, PBMCs were suspended in complete culture medium. The cell suspension was resuscitated in an incubator set at 37 °C under a 5% ${\mathrm{CO}}_{2}$ environment for 1 h. The cells were then centrifuged at 2000 rpm for 5 min. For analysis of Treg, PBMCs were aliquoted into tubes for further staining.

#### 2.3.2 Surface and Intracellular Staining

Treg commonly identified by their expression of CD4 and CD25 on the cell surface and the transcription factor Forkhead box P3 (Foxp3) in the nucleus [23]. For Treg analysis, the cells were incubated with Fluorescein isothiocyanate (FITC) anti-human CD4 and Allophycocyanin (APC) anti-human CD25. After the surface staining, the cells were stained with phycoerythrin (PE) anti-human Foxp3 for Treg detection after fixation and permeabilization. Isotype controls were given to enable correct compensation and confirm antibody specificity. All of the antibodies were from Biolegend. Stained cells were detected by the Attune NxT cytometer and analyzed by the FlowJo 10.0.7.2 software (Reachsoft, Beijing, China).

### 2.4 CMR Protocol and Analysis 

All patients were studied with a 3.0-T scanner (MAGNETOM Singovia; Siemens Healthcare, Erlangen, Germany) within 5–7 days after pPCI. All CMR data were evaluated by two experienced CMR analyst. The scan protocol was performed according to the guidelines of the Society of Cardiovascular Magnetic Resonance [24].

T2-weighted imaging was performed, and myocardium with a signal intensity >2 standard deviation (SD) above the mean signal intensity of remote noninfarcted myocardium was considered the area at risk (AAR). IMH was defined as the hypoenhanced region within the AAR. The CMR instrument used in this study is a 3.0-T scanner. CMR data were transferred to CV142 (Release 5.12.2, Circle Cardiovascular Imaging, Calgary, Canada) software and evaluated twice by an experienced CMR analyst. Another expert in CMR repeated the data evaluation.

### 2.5 Statistical Analysis

Continuous variables are presented as mean ± SD or median (IQR). Comparisons between the study groups were performed by Student’s *t* test or Mann-Whitney U-test. Categorical variables are presented as numbers and percentages, and compared using the Pearson Chi square test. Multivariate logistic regression analysis was used to find the factors that independently predicted IMH. In addition, intercorrelations among variables were taken into consideration in the multivariate analysis. The receiver operating characteristic (ROC) curve analysis was used to evaluate the discriminatory capability of the biomarkers for IMH. 1:1 propensity score matching (PSM) and inverse probability weighting (IPTW) analysis was performed to control the confounding factors. The cutoff value was defined for the maximum Youden index. We also calculated net reclassification improvement (NRI), integrated discrimination improvement (IDI) and C-index to determine the extent to which the addition of new prediction model improves the predictive power of existing baseline risk model. The nomogram was made to calculate the predicted value of an individual suffering from IMH. Data were analyzed using IBM SPSS statistics 24 (Beijing Uone-Tech, Beijing, China), MedCalc 19.1.2 (Reachsoft, Beijing, China) and R Programming Language 4.0.3. *p* value < 0.05 was considered statistically significant.

## 3. Results

### 3.1 Baseline Characteristics of Patients

According to the CMR results, patients were divided into IMH group (n = 80) and no-IMH group (n = 102). The study patients had an average age of 58.0 ± 11.6 years and 150 (82.4%) patients were male. As shown in Table 1, the frequencies of Treg in the IMH group [0.8 (0.3, 1.1)] was significantly lower than that in the no-IMH group [1.3 (0.7, 1.8)] (*p* < 0.001). There were significant differences (*p* < 0.05) between the 2 groups in terms of medical history [angiotensin-converting enzyme inhibitor/angiotensin receptor blocker (ACEI/ARB)], diagnosis at admission (anterior myocardial infarction [MI]), white blood cell (WBC), hsCRP, fasting plasma glucose (FPG), myocardial enzyme [pCKMB, peak value of myoglobin (pMYO) and peak value of troponin I (pTNI)], peak value of N-terminal pro-brain natriuretic peptide (pNT-pro BNP), LVESD and LVEF. No significant difference was found in the other indicators.

**Table 1.** **Clinical characteristics of the 2 groups**.

		Total	No-IMH	IMH	*p* value
		(n = 182)	(n = 102)	(n = 80)	
Lym/PBMCs (%)		69.5 (59.9, 77.3)	69.7 (59.1, 78.6)	69.2 (59.4, 76.3)	0.522
CD4+ T cells/Lym (%)		34.5 (27.3, 45.1)	34.1 (26.5, 45.6)	33.9 (27.6, 45.0)	0.868
Treg/CD4+ T cells (%)		1.0 (0.5, 1.5)	1.3 (0.7, 1.8)	0.8 (0.3, 1.1)	<0.001
Age, years		58.0 ± 11.6	59.1 ± 10.2	56.7 ± 13.1	0.235
Male gender		150 (82.4)	84 (82.4)	66 (82.5)	0.979
BMI, kg/$m^{2}$		25.9 ± 3.6	26.1 ± 3.6	25.7 ± 3.7	0.346
SBP, mmHg		124.9 ± 20.0	125.8 ± 20.8	123.9 ± 19.1	0.628
DBP, mmHg		77.1 ± 13.3	77.3 ± 13.2	76.8 ± 13.5	0.805
Heart rate, bpm		74.8 ± 14.3	73.1 ± 13.9	76.8 ± 14.7	0.191
Medical history					
	Current/ex-smoker	132 (72.5)	73 (71.6)	59 (73.8)	0.744
	Diabetes mellitus	63 (34.6)	34 (33.3)	29 (36.3)	0.681
	Hypertension	119 (65.4)	67 (65.7)	52 (65.0)	0.923
	Stroke	20 (11.0)	11 (10.8)	9 (11.3)	0.921
	Dyslipidemia	139 (76.4)	79 (77.5)	60 (75.0)	0.699
	Antiplatelet agent	16 (8.8)	7 (6.9)	9 (11.3)	0.300
	ACEI/ARB	44 (24.2)	31 (30.4)	13 (16.3)	0.027
	Beta-blocker	16 (8.8)	11 (10.8)	5 (6.3)	0.284
	Statins	12 (6.6)	6 (5.9)	6 (7.5)	0.662
Diagnosis at admission					
	Anterior MI	87 (47.8)	41 (40.2)	46 (57.5)	0.020
In-hospital treatment					
	PCI/CABG	182 (100.0)	102 (100.0)	80 (100.0)	1.000
	Antiplatelet agent	182 (100.0)	102 (100.0)	80 (100.0)	1.000
	ACEI/ARB	79 (43.4)	48 (47.1)	31 (38.8)	0.262
	Beta-blocker	146 (80.2)	79 (77.5)	67 (83.8)	0.290
	Statins	159 (87.4)	93 (91.2)	66 (82.5)	0.080
Hypoglycemic agents					
	Metformin	22 (12.1)	11 (10.8)	11 (13.8)	0.542
	AGI	25 (13.7)	16 (15.7)	9 (11.3)	0.388
	Sulfonylurea	7 (3.8)	4 (3.9)	3 (3.8)	0.952
	DPP-4i	3 (1.6)	2 (2.0)	1 (1.3)	0.709
	SGLT-2i	3 (1.6)	1 (1.0)	2 (2.5)	0.424
	Insulin	8 (4.4)	4 (3.9)	4 (5.0)	0.725
	In-hospital time (d)	8.9 ± 2.6	8.7 ± 2.8	9.3 ± 2.3	0.144
Laboratory values					
	WBC, ${\mathrm{10}}^{9}$/L	9.2 ± 2.6	8.8 ± 2.6	9.7 ± 2.5	0.034
	Hemoglobin, g/L	147.8 ± 15.2	145.7 ± 15.6	150.3 ± 14.3	0.154
	hsCRP, mg/L	4.8 (2.3, 15.4)	3.5 (2.1, 8.7)	8.0 (3.3, 23.2)	<0.001
	FPG, mmol/L	6.3 (5.5, 8.9)	6.0 (5.3, 8.3)	6.7 (5.8, 9.4)	0.009
	RBG, mmol/L	8.4 (7.2, 12.1)	8.2 (7.0, 12.2)	8.6 (7.3, 12.1)	0.465
	HbA1c, %	6.6 ± 1.7	6.4 ± 1.5	6.7 ± 1.9	0.102
	Albumin, g/L	41.7 ± 4.2	41.1 ± 4.8	42.4 ± 3.2	0.053
	ALT, U/L	26.5 (18.0, 38.3)	24.5 (17.0, 34.8)	28.0 (19.3, 39.8)	0.152
	Creatinine, µmol/L	70.8 ± 18.1	70.0 ± 18.3	70.5 ± 18.0	0.663
	eGFR, mL/min/1.73 $m^{2}$	99.5 ± 19.6	98.8 ± 19.2	100.4 ± 19.3	0.441
	TC, mmol/L	5.0 ± 1.1	5.0 ± 1.1	5.0 ± 1.1	0.910
	TGs, mmol/L	1.6 (1.2, 2.4)	1.6 (1.2, 2.5)	1.6 (1.1, 2.2)	0.579
	LDL-C, mmol/L	3.0 ± 0.7	3.0 ± 0.7	3.1 ± 0.7	0.799
	HDL-C, mmol/L	1.00 ± 0.21	1.00 ± 0.21	0.99 ± 0.21	0.832
Myocardial enzyme					
	pCKMB, ng/mL	189 (101, 348)	137 (47, 294)	244 (139, 486)	<0.001
	pMYO, ng/mL	89 (49, 204)	76 (40, 186)	112 (59, 300)	0.039
	pTNI, ng/mL	30 (26, 50)	25 (13, 50)	42 (33, 50)	<0.001
	pNT-pro BNP, pg/mL	1433 (789, 2610)	1131 (576, 2132)	1952 (946, 3162)	0.002
Echocardiography					
	LA, cm	3.81 ± 0.41	3.80 ± 0.43	3.82 ± 0.38	0.813
	LVEDD, cm	5.0 ± 0.4	4.9 ± 0.4	5.1 ± 0.4	0.054
	LVESD, cm	3.5 ± 0.4	3.4 ± 0.4	3.7 ± 0.4	0.002
	LVEF, %	51.1 ± 7.6	52.3 ± 8.1	49.6 ± 6.6	0.034
Angiography findings					
	Multi-vessel/LM	113 (62.1)	65 (63.7)	48 (60.0)	0.607
	Proximal LAD	74 (40.7)	36 (35.3)	38 (47.5)	0.096
	CTO	14 (7.7)	7 (6.9)	7 (8.8)	0.635

IMH, intramyocardial hemorrhage; Lym, Lymphocyte; PBMCs, peripheral blood mononuclear cells; Treg, regulatory T cell; BMI, body mass index; SBP, systolic blood pressure; DBP, diastolic blood pressure; ACEI/ARB, angiotensin-converting enzyme inhibitor/angiotensin receptor blocker; MI, myocardial infarction; PCI/CABG, percutaneous coronary intervention/coronary artery bypass graft; AGI, alpha-glucosidase inhibitor; DPP-4i, dipeptidyl peptidase-4 inhibitor; SGLT-2i, sodium/glucose cotransporter-2 inhibitor; WBC, white blood cell; hsCRP, hypersensitive C-reactive protein; FPG, fasting plasma glucose; RBG, random blood glucose; HbA1c, glycated hemoglobin; ALT, alanine transaminase; eGFR, estimated glomerular filtration rate; TC, total cholesterol; TGs, triglycerides; LDL-C, low-density lipoprotein cholesterol; HDL-C, high-density lipoprotein cholesterol; pCKMB, the peak value of creatine kinase MB; pMYO, the peak value of myoglobin; pTNI, the peak value of troponin I; NT-pro BNP, N-terminal pro-brain natriuretic peptide; LA, left atrium; LVEDD, left ventricular end-diastolic diameter; LVESD, left ventricular end-systolic diameter; LVEF, left ventricular ejection fraction; LM, left main coronary artery; LAD, left anterior descending; CTO, chronic total occlusions.

**Fig. 1.**
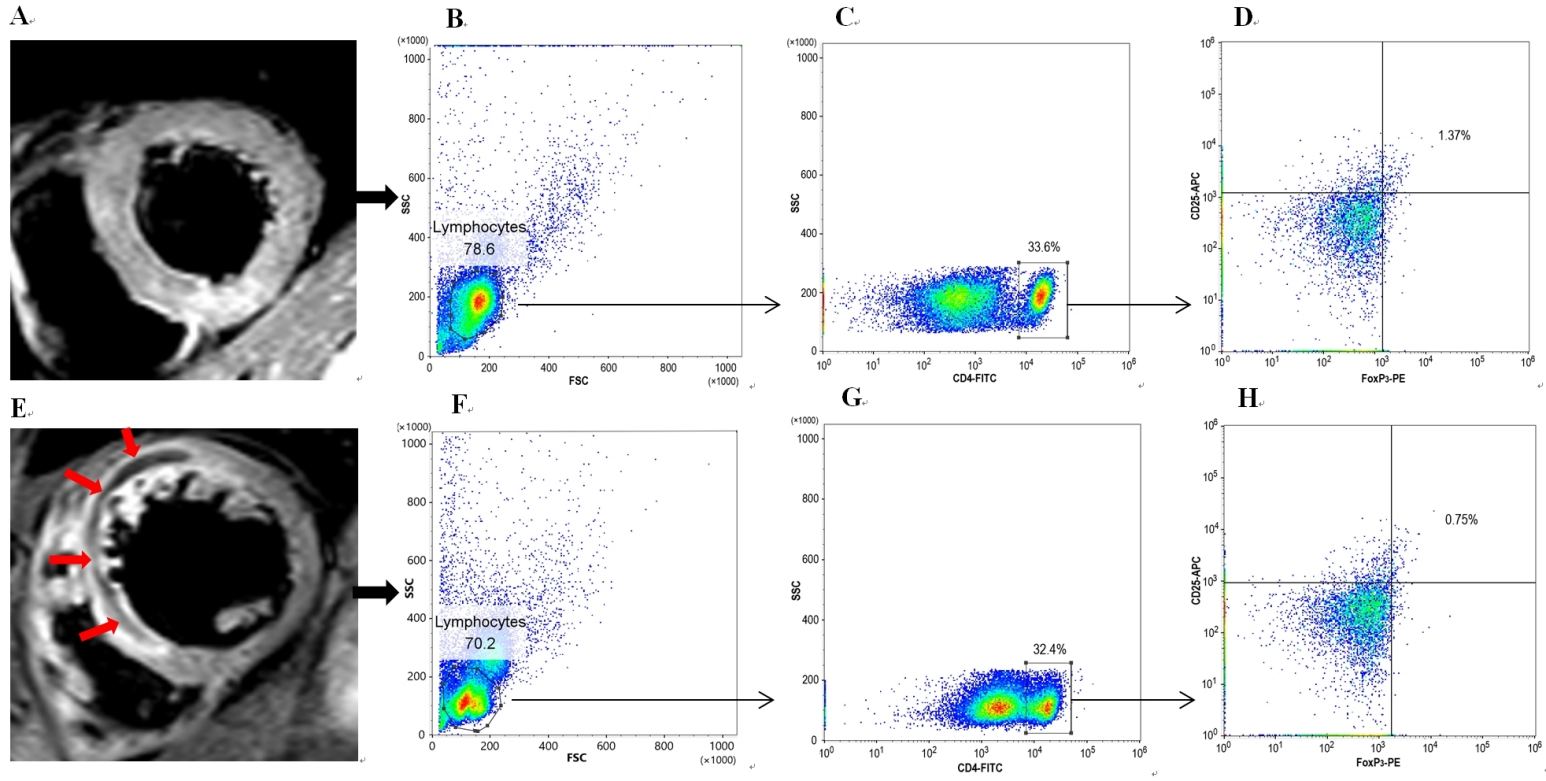
** CMR examples with or without IMH burden and their circulating Treg frequencies**. Panels (A–D) show a case without IMH burden who is diagnosed as acute anterior myocardial infarction. Panels (E–H) show a case with IMH burden who is also diagnosed as acute anterior myocardial infarction. The red arrows in panel (E) represent the region of IMH. According to panels (D) and (H), the Treg frequency in patients with IMH (0.75%) is significantly lower than that in patients without IMH (1.37%). CMR, cardiac magnetic resonance imaging; IMH, intramyocardial hemorrhage; Treg, regulatory T cell.

Fig. 1 showed the CMR images and flow scatter diagrams of representative cases of STEMI in patients with IMH group and no-IMH group, respectively. The hypoenhanced region in “panel E” (red arrows) was the area of IMH.

### 3.2 Predictors of IMH

Table 2 showed the results of the univariate and multivariate logistic regression analyses. In univariate analysis, decreased Treg frequency, increased hsCRP, pCKMB and LVESD were significantly associated with IMH, as were decreased LVEF, no previous use of ACEI/ARB, anterior MI diagnosed at admission, increased WBC, FPG and pTNI. Correlation analysis showed that pCKMB was significantly correlated with pTNI (r = 0.623, *p* < 0.001). In addition, hsCRP was correlated with WBC (r = 0.684, *p* < 0.001). Therefore, pTNI and WBC were not included in the multivariate model. After multivariable adjustment, decreased Treg frequency [odds ratio (OR) (95% confidence interval (CI)): 0.350 (0.202–0.606), *p* < 0.001], increased hsCRP [OR (95% CI): 1.060 (1.022–1.100), *p* = 0.002], pCKMB [OR (95% CI): 1.004 (1.001–1.006), *p* = 0.002] and LVESD [OR (95% CI): 3.329 (1.346–8.236), *p* = 0.009] were determined to be independent predictors of IMH in STEMI patients received PPCI.

**Table 2.** **Univariate and multivariate logistic regression analysis of IMH occurrence in patients with STEMI-PPCI**.

		Univariate	*p* value	Multivariate	*p* value
		OR (95% CI)		Adjusted OR (95% CI)	
Treg/CD4+ T cells (%)		0.387 (0.245, 0.613)	<0.001	0.350 (0.202, 0.606)	<0.001
Age, years		0.985 (0.960, 1.010)	0.234		
Male gender		1.010 (0.468, 2.179)	0.979		
Medical history					
	ACEI/ARB	0.444 (0.214, 0.921)	0.029	0.504 (0.213, 1.192)	0.119
Diagnosis at admission					
	Anterior MI	2.012 (1.111, 3.650)	0.021	2.066 (0.962, 4.425)	0.063
	WBC, ${\mathrm{10}}^{9}$/L	1.128 (1.008, 1.263)	0.036		
	hsCRP, mg/L	1.048 (1.019, 1.078)	0.001	1.060 (1.022, 1.100)	0.002
	FPG, mmol/L	1.141 (1.027, 1.267)	0.014	1.042 (0.915, 1.187)	0.537
Myocardial enzyme					
	pCKMB, ng/mL	1.004 (1.002, 1.006)	<0.001	1.004 (1.001, 1.006)	0.002
	pMYO, ng/mL	1.001 (1.000, 1.003)	0.123		
	pTNI, ng/mL	1.105 (1.063, 1.149)	<0.001		
	pNT-pro BNP, pg/mL	1.001 (0.998, 1.002)	0.261		
Echocardiography					
	LVESD, cm	2.978 (1.438, 6.165)	0.003	3.329 (1.346, 8.236)	0.009
	LVEF, %	0.960 (0.923, 0.997)	0.037	1.054 (0.996, 1.115)	0.069

IMH, intramyocardial hemorrhage; STEMI, ST-segment elevation myocardial infarction; PPCI, primary percutaneous coronary intervention; OR, odds ratio; CI, confidence interval; Treg, regulatory T cell; ACEI/ARB, angiotensin-converting enzyme inhibitor/angiotensin receptor blocker; MI, myocardial infarction; WBC, white blood cell; hsCRP, hypersensitive C-reactive protein; FPG, fasting plasma glucose; pCKMB, the peak value of creatine kinase MB; pMYO, the peak value of myoglobin; pTNI, the peak value of troponin I; NT-pro BNP, N-terminal pro-brain natriuretic peptide; LVESD, left ventricular end-systolic diameter; LVEF, left ventricular ejection fraction.

In addition, IPTW was also used to assess the predictive effect of the above risk factors on the occurrence of IMH. IPTW analysis also showed that Treg frequency [OR (95% CI): 0.371 (0.217–0.635), *p* < 0.001], hsCRP [OR (95% CI): 1.052 (1.016–1.089), *p* = 0.004], pCKMB [OR (95% CI): 1.003 (1.001–1.006), *p* = 0.003] and LVESD [OR (95% CI): 2.431 (1.089–5.427), *p* = 0.030] were determined to be independent predictors of IMH.

### 3.3 ROC Curve Analysis of 4 Predictors

**Fig. 2.**
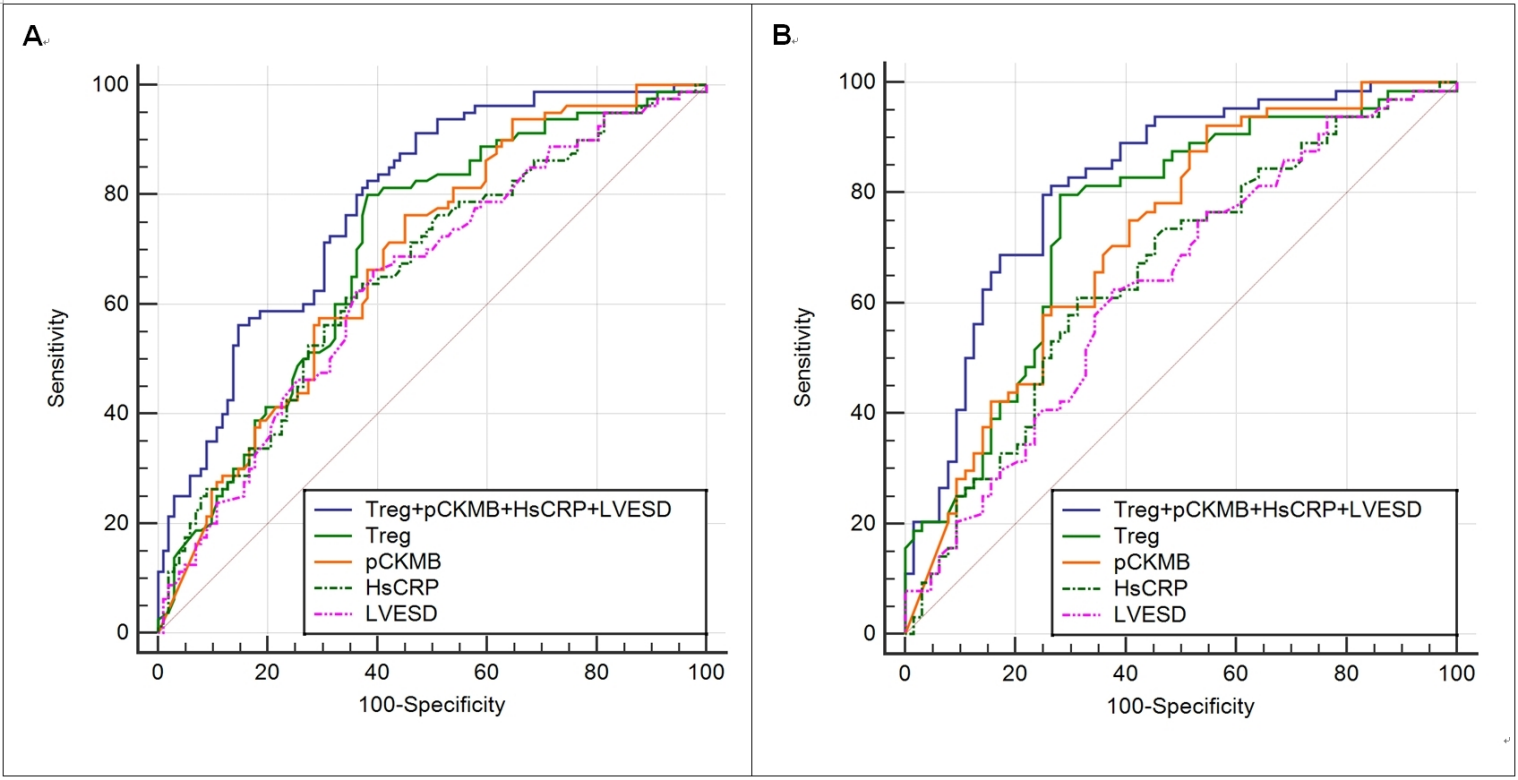
**Pre-(A) and postmatching (B) ROC curve analysis of Treg, pCKMB, hsCRP and LVESD for the prediction of IMH**. Treg, regulatory T cell; hsCRP, hypersensitive C-reactive protein; pCKMB, the peak value of creatine kinase MB; LVESD, left ventricular end-systolic diameter; IMH, intramyocardial hemorrhage; ROC, the receiver operating characteristic.

Before PSM, the results of the ROC analysis detailed in Table 3 and Fig. 2 revealed that all 4 biomarkers significantly predicted the presence of IMH (area under the ROC curve [AUC]: Treg 0.701, pCKMB 0.684, hsCRP 0.658, and LVESD 0.646; all *p <* 0.01). According to the maximum Youden indexes, the cutoff values for Treg, pCKMB, hsCRP, and LVESD were 1.07%, 137.5 ng/mL, 5.74 mg/L, and 3.52 cm, respectively. The AUC for the combination of Treg, pCKMB, hsCRP, and LVESD was 0.786 (*p <* 0.001), indicating very good discriminative ability for the prediction of IMH. Notably, the discriminatory capability for IMH of the 4-biomarker panel was stronger than those of the individual biomarkers (*p < 0.05).

**Table 3.** **Pre- and postmatching receiver operating characteristic curve analysis of Treg, hsCRP, pCKMB and LVESD for the prediction of IMH**.

		Cutoff value	AUC	95% CI	*p* value	Sensitivity	Specificity	Youden index
Pre-matching								
	LVESD, cm	3.52	0.646*	0.566–0.726	0.001	0.663	0.608	0.270
	hsCRP, mg/L	5.74	0.658*	0.579–0.738	<0.001	0.613	0.657	0.270
	pCKMB, ng/mL	137.5	0.684*	0.608–0.761	<0.001	0.763	0.549	0.312
	Treg, %	1.07	0.701*	0.625–0.777	<0.001	0.800	0.618	0.418
	Treg + pCKMB + hsCRP + LVESD	-/-	0.786	0.721–0.851	<0.001	0.825	0.618	0.443
Post-matching								
	LVESD, cm	3.52	0.633*	0.537–0.729	0.009	0.625	0.625	0.250
	hsCRP, mg/L	5.20	0.656*	0.561–0.751	0.002	0.609	0.687	0.297
	pCKMB, ng/mL	73.2	0.721*	0.633–0.809	<0.001	0.922	0.453	0.375
	Treg, %	1.07	0.750	0.663–0.836	<0.001	0.797	0.719	0.516
	Treg + pCKMB + hsCRP + LVESD	-/-	0.821	0.747–0.894	<0.001	0.813	0.734	0.547

Note: Compared with Treg + pCKMB + hsCRP + LVESD *p < 0.05. Treg, regulatory T cell; hsCRP, hypersensitive C-reactive protein; pCKMB, the peak value of creatine kinase MB; LVESD, left ventricular end-systolic diameter; IMH, intramyocardial hemorrhage; CI, confidence interval; AUC, area under the ROC curve.

After PSM, the age, gender, BMI, medical history (ACEI/ARB), diagnosis at admission (anterior MI), and FPG were not statistically different between the 2 groups. ROC analysis (Table 3, Fig. 2) still showed that the discriminatory capability of the 4-biomarker panel was good (AUC 0.821, 95% CI 0.747–0.894) and stronger than that of the pCKMB, hsCRP and LVESD [with AUC values of 0.721 for pCKMB, 0.656 for hsCRP, and 0.633 for LVESD] (*p < 0.05). The AUC value of Treg was 0.750, and there is no significant difference in the discriminatory capability for IMH between Treg and 4-biomarker panel. The maximum Youden indexes showed that the cutoff values for Treg, pCKMB, hsCRP and LVESD were 1.07%, 73.2 ng/mL, 5.20 mg/L, and 3.52 cm, respectively.

### 3.4 Incremental Effect of 4 Predictors on Predictive Value for IMH

**Fig. 3.**
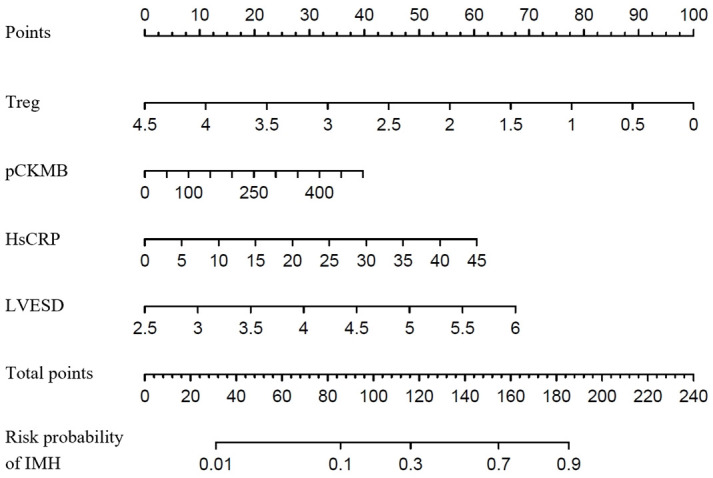
**The proposed nomogram for predicting the risk probability of IMH**. Treg, regulatory T cell; hsCRP, hypersensitive C-reactive protein; pCKMB, the peak value of creatine kinase MB; LVESD, left ventricular end-systolic diameter; IMH, intramyocardial hemorrhage.

Table 4 showed that compared with the LVESD, hsCRP, pCKMB and Treg, the addition of the combined index (Treg + pCKMB + hsCRP + LVESD) significantly improved the reclassification and discrimination ability beyond the baseline risk model with NRI of 0.197, and IDI of 0.200 (both *p* < 0.05). In addition, the C-index of the baseline risk model changed after addition of the combined index [0.806 (0.744 to 0.869), *p* < 0.001]. The nomogram in Fig. 3 was used to calculate the predicted value of an individual suffering from IMH.

**Table 4.** **Evaluate the predictive power and incremental predictive value of various models with NRI, IDI and C-index**.

	Category-free NRI			IDI			C-index		
	Index	95% CI	*p* value	Index	95% CI	*p* value	Index	95% CI	*p* value
Baseline risk model			Ref.			Ref.	0.661	0.580 to 0.741	<0.001
+LVESD	0.046	–0.068 to 0.161	0.428	0.037	0.010 to 0.064	0.008	0.695	0.617 to 0.772	<0.001
+hsCRP	0.058	–0.101 to 0.217	0.476	0.027	0.003 to 0.052	0.030	0.685	0.607 to 0.763	<0.001
+pCKMB	0.195	0.023 to 0.368	0.027	0.067	0.030 to 0.103	<0.001	0.725	0.651 to 0.799	<0.001
+Treg	0.178	0.005 to 0.352	0.044	0.093	0.053 to 0.132	<0.001	0.747	0.677 to 0.181	<0.001
+Combined	0.197	0.039 to 0.356	0.015	0.200	0.142 to 0.259	<0.001	0.806	0.744 to 0.869	<0.001

Baseline risk model including diagnosis at admission (anterior myocardial infarction), FPG, ACEI/ARB used before admission and LVEF. Combined index represents Treg, pCKMB, hsCRP combined with LVESD. NRI, net reclassification improvement; IDI, integrated discrimination improvement; CI, confidence interval; Treg, regulatory T cell; ACEI/ARB, angiotensin-converting enzyme inhibitor/angiotensin receptor blocker; hsCRP, hypersensitive C-reactive protein; FPG, fasting plasma glucose; pCKMB, the peak value of creatine kinase MB; LVESD, left ventricular end-systolic diameter; LVEF, left ventricular ejection fraction.

### 3.5 Association between Number of Abnormal Biomarker Levels and IMH

**Fig. 4.**
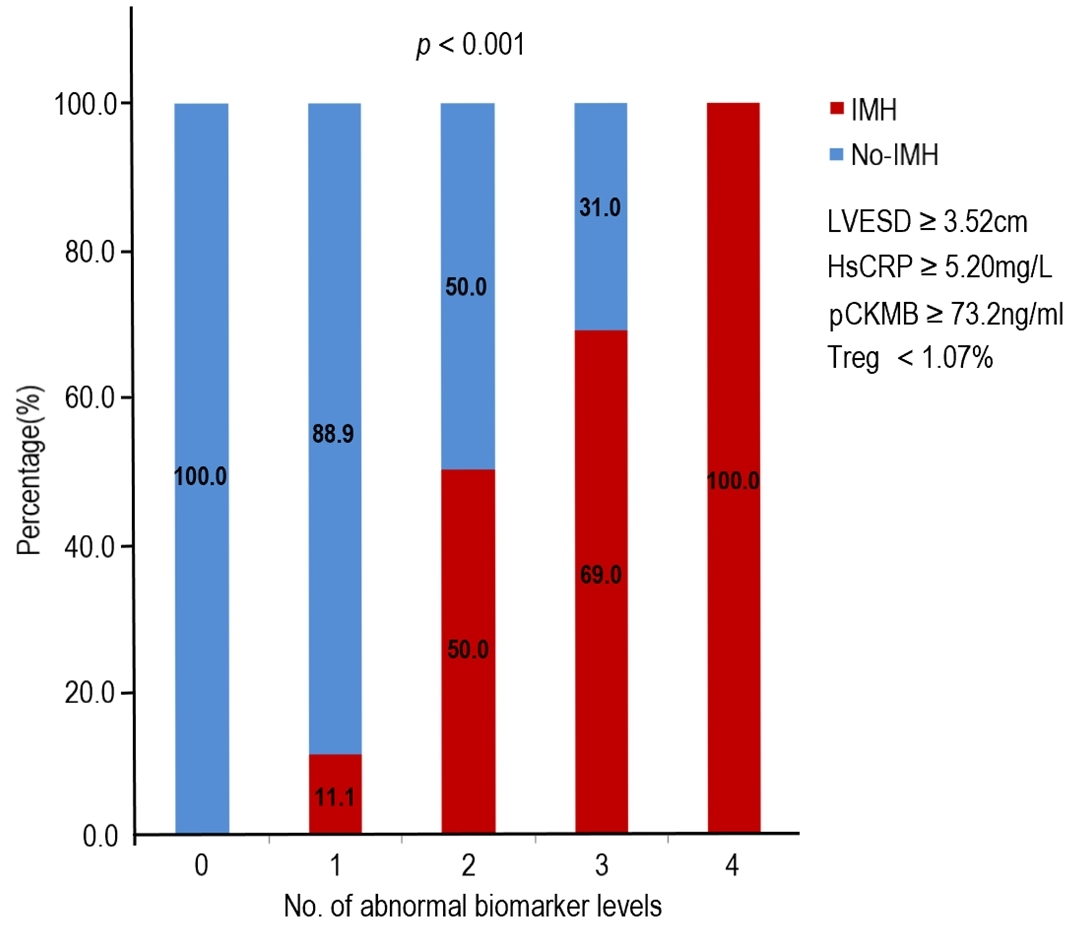
**Association of the number of abnormal biomarker levels based on the identified cutoff values and the risk of IMH**. Treg, regulatory T cell; hsCRP, hypersensitive C-reactive protein; pCKMB, the peak value of creatine kinase MB; LVESD, left ventricular end-systolic diameter; IMH, intramyocardial hemorrhage.

Fig. 4 shows the relationship between the risk of IMH and the number of abnormal biomarker levels. Based on the cutoff value, biomarker levels are defined as normal or abnormal. The risk of IMH increased with the number of abnormal biomarker. The odds of IMH were increased by 11-fold or 39-fold respectively, if patients presented with abnormal levels of 2 or ≥3 biomarkers compared with ≤1 biomarkers (Table 5).

**Table 5.** **Logistic regression analysis of the number of abnormal biomarker and the probability of IMH**.

No.of Abnormal Biomarkers	No-IMH	IMH	OR (95% CI)	*p* value
≤1	35 (92.1)	3 (7.9)	1	-/-
2	16 (50.0)	16 (50.0)	11.7 (3.0–45.8)	<0.001
≥3	13 (22.4)	45 (77.6)	40.4 (10.7–152.8)	<0.001

Cutoff values for abnormal biomarker levels were LVESD ≥3.52 cm, hsCRP ≥5.20 mg/L, pCKMB ≥73.2 ng/mL, and Treg <1.07%. IMH, intramyocardial hemorrhage; OR, odds ratio; CI, confidence interval; pCKMB, the peak value of creatine kinase MB; LVESD, left ventricular end-systolic diameter; hsCRP, hypersensitive C-reactive protein.

## 4. Discussion

This study firstly explored the predictive ability of Treg individually or in combination with other 3 biomarkers, including pCKMB, hsCRP and LVESD, for IMH in STEMI patients underwent PPCI. The major findings were these: (1) in STEMI patients received PPCI, IMH group had lower level of Treg frequency than no-IMH group; (2) Treg frequency, pCKMB, hs-CRP and LVESD were independent risk factors for IMH; (3) the discriminatory capability of the 4-biomarker panel (combination of Treg, pCKMB, hsCRP and LVESD) for IMH was stronger than those of the biomarker individually, except Treg; and (4) increased number of abnormal biomarkers led to a significant increase in the risk of IMH (Treg <1.07%, pCKMB ≥73.2 ng/mL, hsCRP ≥5.20 mg/L, and LVESD ≥3.52 cm). Based on the results of this study, we confirmed the effectiveness of Treg individually or in combination with pCKMB, hsCRP and LVESD in predicting IMH. For those patients who cannot undergo CMR examination, Treg undoubtedly is a simple, safe and effective method to predict IMH in clinical practice.

Acute myocardial infarction, including non-ST elevated myocardial infarction and STEMI, is the leading cause of cardiovascular disease and mortality worldwide [25]. In STEMI, the coronary artery is often acutely completely blocked, and it is pivotal to open the infarct-related artery (IRA) urgently. Therefore, the preferred treatment strategy for STEMI patients within 12 h of symptom onset is coronary revascularization by PPCI in combination with antithrombotic therapy [1, 2]. However, despite the IRA’s epicardial flow has been restored, a sizable proportion of patients continue to experience hypoperfusion of myocardial tissue after PPCI, called no-reflow phenomenon. IMH is a form of no-reflow phenomenon resulting from the extensive erythrocyte aggregation and extravasation because of the damaged endothelial walls [5, 6]. IMH can be visualized by T2-weighted CMR because breakdown products of hemoglobin are paramagnetic and influence regional magnetic tissue properties [26]. However, not all patients are suitable for CMR to clarify the presence of IMH, such as those with serious condition or contraindications. Therefore, we urgently need to find a simple and effective method to predict the presence of IMH. Carrick* et al*. [11] revealed that IMH was a better predictor of adverse events after myocardial infarction than MVO. Husser* et al*. [27] found that the incidence of MACEs was significantly higher in the IMH group than in the non-IMH group. In addition, the Kaplan-Meier analysis showed that the MACE-free survival was significant prolonged in patients without IMH, and IMH is a predictor of poor myocardial remodeling [27]. In addition, Amier* et al*. [3] also demonstrated that IMH was associated with larger myocardial infarct size, greater MVO range, and lower LVEF. Therefore, in this study, we analyzed various indexes of myocardial enzymes and left ventricular structure, and found that pCKMB and LVESD were independent predictors of IMH. Although the molecular mechanisms involved in the initiation and progression of MIRI are still not completely understood, accumulating evidences have suggested that excessive inflammation plays a predominant role in it [15, 28]. And Carrick* et al*. [11] also found that IMH was associated with markers of inflammation, including peak monocyte count and peak neutrophil count. In this study, we also found that inflammatory markers were significantly higher in patients with IMH than in patients without IMH, and hsCRP was an independent predictor of IMH. Considering that Treg cells have been verified to playing an anti-inflammatory effect in MIRI by inhibiting the macrophage inflammatory phenotype and neutrophil function in animal experiments [18, 21, 22], we hypothesized that the circulating Treg levels are related to the presence of IMH in STEMI patients. The final results were consistent with our expectation that Treg is a protective factor of IMH and can independently predict IMH occurrence in STEMI patients received PPCI. Moreover, we proposed for the first time that Treg can be combined with pCKMB, hsCRP and LVESD to predict the presence of IMH. These findings not only indirectly indicated that Treg may have a certain protective effect on human MIRI, but also provided us with a simpler method to predict IMH than CMR, especially for those who can’t finish the CMR. Considering that previous studies have reported that adoptive transfer of Tregs is beneficial in kidney, brain, liver and myocardial ischaemia/reperfusion (I/R) injury [19, 20, 29, 30], we can assume that adoptive transfer of Tregs can significantly reduce the occurrence of IMH in STEMI patients received PPCI. In the future, we will do a lot of work to confirm the scientific hypothesis that adoptive transfer of Tregs may be an effective biologic therapy for the prevention or treatment of IMH after myocardial infarction.

Limitations of the study are as follows. First, it was a single-center study with a small sample size. Therefore, the research results obtained need to be further verified by a large sample size and multi-center study. Second, because the laboratory parameters are measured only once, there may be potential bias due to measurement error. Third, this study did not include the follow-up data, and it is not clear whether Treg levels can predict prognosis of STEMI patients. In future work, we will not only continue to expand the sample size to confirm the stability of the conclusions, but also collect the follow-up data to further explore the impact of Treg level on the prognosis.

## 5. Conclusions

In a word, the current study firstly showed that Treg individually or in combination with pCKMB, hsCRP, and LVESD can effectively predict the presence of IMH in STEMI-PPCI patients.

## Data Availability

The datasets used and/or analyzed during the current study are available from the corresponding author on reasonable request.

## Abbreviations

IMH, intramyocardial hemorrhage; STEMI, ST-segment elevation myocardial infarction; PPCI, primary percutaneous coronary intervention; Treg, regulatory T cell; pCKMB, the peak value of Creatine Kinase MB; hsCRP, high-sensitivity C-reactive protein; LVESD, left ventricular end-systolic diameter; NRI, net reclassification improvement; IDI, integrated discrimination improvement; PSM, propensity score matching; IPTW, inverse probability weighting; TIMI, thrombolysis in myocardial infarction flow; MVO, microvascular obstruction; LV, left ventricular; MACEs, major adverse cardiac events; CMR, cardiac magnetic resonance; MIRI, myocardial ischaemia/reperfusion injury; IRI, ischaemia-reperfusion injury; Th1, T-helper 1; Th2, T-helper 2; Th17, T-helper 17; CABG, coronary artery bypass graft; LVEF, left ventricular ejection fraction; eGFR, estimated glomerular filtration rate; PBMCs, peripheral blood mononuclear cells; Foxp3, transcription factor Forkhead box P3; FITC, fluorescein isothiocyanate; APC, allophycocyanin; PE, phycoerythrin; SD, standard deviation; AAR, area at risk; ROC, receiver operating characteristic; ACEI/ARB, angiotensin-converting enzyme inhibitor/angiotensin receptor blocker; WBC, white blood cell; FPG, fasting plasma glucose; pMYO, the peak value of myoglobin; pTNI, the peak value of troponin I; pNT-pro BNP, the peak value of N-terminal pro-brain natriuretic peptide; AUC, area under the ROC curve; IRA, infarct-related artery.

## Supplementary Material

Supplementary material associated with this article can be found, in the online version, at https://doi.org/10.31083/j.rcm2407205.

## References

[1] Ibanez B, James S, Agewall S, Antunes MJ, Bucciarelli-Ducci C, Bueno H (2018). 2017 ESC Guidelines for the management of acute myocardial infarction in patients presenting with ST-segment elevation: The Task Force for the management of acute myocardial infarction in patients presenting with ST-segment elevation of the European Society of Cardiology (ESC). *European Heart Journal*.

[2] O’Gara PT, Kushner FG, Ascheim DD, Casey DE, Chung MK, de Lemos JA (2013). 2013 ACCF/AHA guideline for the management of ST-elevation myocardial infarction: executive summary: a report of the American College of Cardiology Foundation/American Heart Association Task Force on Practice Guidelines. *Circulation*.

[3] Amier RP, Tijssen RYG, Teunissen PFA, Fernández-Jiménez R, Pizarro G, García-Lunar I (2017). Predictors of Intramyocardial Hemorrhage After Reperfused ST-Segment Elevation Myocardial Infarction. *Journal of the American Heart Association*.

[4] Symons R, Pontone G, Schwitter J, Francone M, Iglesias JF, Barison A (2018). Long-Term Incremental Prognostic Value of Cardiovascular Magnetic Resonance After ST-Segment Elevation Myocardial Infarction: A Study of the Collaborative Registry on CMR in STEMI. *JACC: Cardiovascular Imaging*.

[5] Betgem RP, de Waard GA, Nijveldt R, Beek AM, Escaned J, van Royen N (2015). Intramyocardial haemorrhage after acute myocardial infarction. *Nature Reviews. Cardiology*.

[6] Robbers LFHJ, Eerenberg ES, Teunissen PFA, Jansen MF, Hollander MR, Horrevoets AJG (2013). Magnetic resonance imaging-defined areas of microvascular obstruction after acute myocardial infarction represent microvascular destruction and haemorrhage. *European Heart Journal*.

[7] Eitel I, Kubusch K, Strohm O, Desch S, Mikami Y, de Waha S (2011). Prognostic value and determinants of a hypointense infarct core in T2-weighted cardiac magnetic resonance in acute reperfused ST-elevation-myocardial infarction. *Circulation. Cardiovascular Imaging*.

[8] Ganame J, Messalli G, Dymarkowski S, Rademakers FE, Desmet W, Van de Werf F (2009). Impact of myocardial haemorrhage on left ventricular function and remodelling in patients with reperfused acute myocardial infarction. *European Heart Journal*.

[9] Beek AM, Nijveldt R, van Rossum AC (2010). Intramyocardial hemorrhage and microvascular obstruction after primary percutaneous coronary intervention. *The International Journal of Cardiovascular Imaging*.

[10] Reinstadler SJ, Stiermaier T, Reindl M, Feistritzer H, Fuernau G, Eitel C (2019). Intramyocardial haemorrhage and prognosis after ST-elevation myocardial infarction. *European Heart Journal. Cardiovascular Imaging*.

[11] Carrick D, Haig C, Ahmed N, McEntegart M, Petrie MC, Eteiba H (2016). Myocardial Hemorrhage After Acute Reperfused ST-Segment-Elevation Myocardial Infarction: Relation to Microvascular Obstruction and Prognostic Significance. *Circulation. Cardiovascular Imaging*.

[12] Hamirani YS, Wong A, Kramer CM, Salerno M (2014). Effect of microvascular obstruction and intramyocardial hemorrhage by CMR on LV remodeling and outcomes after myocardial infarction: a systematic review and meta-analysis. *JACC: Cardiovascular Imaging*.

[13] Ibanez B, Aletras AH, Arai AE, Arheden H, Bax J, Berry C (2019). Cardiac MRI Endpoints in Myocardial Infarction Experimental and Clinical Trials: JACC Scientific Expert Panel. *Journal of the American College of Cardiology*.

[14] Niccoli G, Scalone G, Lerman A, Crea F (2016). Coronary microvascular obstruction in acute myocardial infarction. *European Heart Journal*.

[15] Timmers L, Pasterkamp G, de Hoog VC, Arslan F, Appelman Y, de Kleijn DPV (2012). The innate immune response in reperfused myocardium. *Cardiovascular Research*.

[16] Jaffe R, Dick A, Strauss BH (2010). Prevention and treatment of microvascular obstruction-related myocardial injury and coronary no-reflow following percutaneous coronary intervention: a systematic approach. *JACC: Cardiovascular Interventions*.

[17] Bochaton T, Lassus J, Paccalet A, Derimay F, Rioufol G, Prieur C (2021). Association of myocardial hemorrhage and persistent microvascular obstruction with circulating inflammatory biomarkers in STEMI patients. *PLoS ONE*.

[18] Mahajan D, Wang Y, Qin X, Wang Y, Zheng G, Wang YM (2006). CD4+CD25+ regulatory T cells protect against injury in an innate murine model of chronic kidney disease. *Journal of the American Society of Nephrology: JASN*.

[19] Kinsey GR, Sharma R, Huang L, Li L, Vergis AL, Ye H (2009). Regulatory T cells suppress innate immunity in kidney ischemia-reperfusion injury. *Journal of the American Society of Nephrology: JASN*.

[20] Li P, Gan Y, Sun B, Zhang F, Lu B, Gao Y (2013). Adoptive regulatory T-cell therapy protects against cerebral ischemia. *Annals of Neurology*.

[21] Xia N, Jiao J, Tang T, Lv B, Lu Y, Wang K (2015). Activated regulatory T-cells attenuate myocardial ischaemia/reperfusion injury through a CD39-dependent mechanism. *Clinical Science (London, England: 1979)*.

[22] Xiao J, Yu K, Li M, Xiong C, Wei Y, Zeng Q (2017). The IL-2/Anti-IL-2 Complex Attenuates Cardiac Ischaemia-Reperfusion Injury Through Expansion of Regulatory T Cells. *Cellular Physiology and Biochemistry: International Journal of Experimental Cellular Physiology, Biochemistry, and Pharmacology*.

[23] Sakaguchi S, Ono M, Setoguchi R, Yagi H, Hori S, Fehervari Z (2006). Foxp3+ CD25+ CD4+ natural regulatory T cells in dominant self-tolerance and autoimmune disease. *Immunological Reviews*.

[24] Kramer CM, Barkhausen J, Flamm SD, Kim RJ, Nagel E, Society for Cardiovascular Magnetic Resonance Board of Trustees Task Force on Standardized Protocols (2008). Standardized cardiovascular magnetic resonance imaging (CMR) protocols, society for cardiovascular magnetic resonance: board of trustees task force on standardized protocols. *Journal of Cardiovascular Magnetic Resonance: Official Journal of the Society for Cardiovascular Magnetic Resonance*.

[25] Benjamin EJ, Virani SS, Callaway CW, Chamberlain AM, Chang AR, Cheng S (2018). Heart Disease and Stroke Statistics-2018 Update: A Report From the American Heart Association. *Circulation*.

[26] Lotan CS, Bouchard A, Cranney GB, Bishop SP, Pohost GM (1992). Assessment of postreperfusion myocardial hemorrhage using proton NMR imaging at 1.5 T. *Circulation*.

[27] Husser O, Monmeneu JV, Sanchis J, Nunez J, Lopez-Lereu MP, Bonanad C (2013). Cardiovascular magnetic resonance-derived intramyocardial hemorrhage after STEMI: Influence on long-term prognosis, adverse left ventricular remodeling and relationship with microvascular obstruction. *International Journal of Cardiology*.

[28] Zhang X, Du Q, Yang Y, Wang J, Dou S, Liu C (2017). The protective effect of Luteolin on myocardial ischemia/reperfusion (I/R) injury through TLR4/NF-κB/NLRP3 inflammasome pathway. *Biomedicine & Pharmacotherapy*.

[29] Lu L, Li G, Rao J, Pu L, Yu Y, Wang X (2009). In vitro induced CD4(+)CD25(+)Foxp3(+) Tregs attenuate hepatic ischemia-reperfusion injury. *International Immunopharmacology*.

[30] Tang T, Yuan J, Zhu Z, Zhang W, Xiao H, Xia N (2012). Regulatory T cells ameliorate cardiac remodeling after myocardial infarction. *Basic Research in Cardiology*.

