# Supplementary material for: Regulatory T Cell as Predictor of Intramyocardial Hemorrhage in STEMI Patients after Primary PCI
Source: Rev Cardiovasc Med. 2023 Jul 14;24(7):205. doi: 10.31083/j.rcm2407205 (PMC11266489; doi:10.31083/j.rcm2407205)
Supplement: Supplementary file 1 [file 2153-8174-24-7-205-s1.zip › 2153-8174-24-7-205-s1.docx]

| **Supplementary Table. Baseline CMR characteristics of 2 groups(according to the cutoff value of Treg).** | | | | |
| --- | --- | --- | --- | --- |
|  | Total | Treg<1.07% | Treg≥1.07% | *p* value |
|  | (n=182) | (n=104) | (n=78) |  |
| Treg/CD4+ T cells(%) | 1.0(0.4,1.5) | 0.6(0.2,0.9) | 1.7(1.3,2.1) | <0.001 |
| CMR baseline characteristics |  |  |  |  |
| LVEDV, ml/m^2^ | 143.5±33.5 | 148.8±35.6 | 135.1±28.5 | 0.005 |
| LVESV, ml/m^2^ | 69.2(52.0,93.0) | 76.2(58.1,97.6) | 59.5(44.0,77.3) | <0.001 |
| LVEF, % | 49.9±11.7 | 47.3±11.5 | 53.8±11.1 | 0.001 |
| LV mass, g | 114.9±26.3 | 116.2±27.7 | 112.2±24.0 | 0.227 |
| LGE mass, g | 34.0(19.2,55.2) | 46.3(24.6,65.4) | 24.5(11.7,43.3) | <0.001 |
| LGE, % LV mass | 31.7(17.2,47.1) | 39.3(24.1,52.5) | 21.8(10.4,38.1) | <0.001 |
| AAR, g | 40.2(25.0,54.2) | 42.1(26.8,60.1) | 37.3(22.6,47.8) | 0.020 |
| AAR, % LV mass | 36.5(23.7,48.2) | 39.9(24.8,50.7) | 33.0(20.4,39.9) | 0.017 |
| IMH, prevalence | 80(44.0) | 64(61.5) | 16(20.5) | <0.001 |
| MVO, prevalence | 109(59.9) | 91(87.5) | 18(23.1) | <0.001 |
| MVO mass, g | 2.6(0.9,6.1) | 3.0(1.0,6.2) | 1.3(0.1,4.5) | 0.042 |
| MVO mass, % LV mass | 2.4(0.8,4.7) | 2.6(1.0,5.2) | 1.5(0.1,3.7) | 0.075 |
| MVO mass, % LGE mass | 5.9(1.9,9.4) | 5.9(2.8,10.8) | 5.9(0.3,9.0) | 0.167 |

CMR, cardiac magnetic resonance; Treg, regulatory T cell; LVEDV, left ventricular end-diastolic volume; LVESV, left ventricular end-systolic volume; LVEF, left ventricular ejection fraction; LV, left ventricular; LGE, late gadolinium enhancement; AAR, area at risk; IMH, intramyocardial hemorrhage; MVO, microvascular obstruction.
